# Surface Coating of Oxide Powders: A New Synthesis Method to Process Biomedical Grade Nano-Composites

**DOI:** 10.3390/ma7075012

**Published:** 2014-07-04

**Authors:** Paola Palmero, Laura Montanaro, Helen Reveron, Jérôme Chevalier

**Affiliations:** 1 Department of Applied Science and Technology, Politecnico di Torino, INSTM R.U. PoliTO, Laboratorio di Tecnologia ed Ingegnerizzazione dei Materiali Ceramici (LINCE), Corso Duca degli Abruzzi, 24, Torino 10129, Italy; E-Mail: laura.montanaro@polito.it; 2 Université de Lyon, INSA–Lyon, MATEIS UMR CNRS 5510, Bât. Blaise Pascal 7, Av. Jean Capelle, Villeurbanne 69621, France; E-Mails: helen.reveron@insa-lyon.fr (H.R.); jerome.chevalier@insa-lyon.fr (J.C.); 3 Institut Universitaire de France, 103 bd Saint-Michel, Paris 75005, France

**Keywords:** ceramic composites, biomedical applications, elaboration method, dental and orthopedic applications, alumina-zirconia composites

## Abstract

Composite and nanocomposite ceramics have achieved special interest in recent years when used for biomedical applications. They have demonstrated, in some cases, increased performance, reliability, and stability *in vivo*, with respect to pure monolithic ceramics. Current research aims at developing new compositions and architectures to further increase their properties. However, the ability to tailor the microstructure requires the careful control of all steps of manufacturing, from the synthesis of composite nanopowders, to their processing and sintering. This review aims at deepening understanding of the critical issues associated with the manufacturing of nanocomposite ceramics, focusing on the key role of the synthesis methods to develop homogeneous and tailored microstructures. In this frame, the authors have developed an innovative method, named “surface-coating process”, in which matrix oxide powders are coated with inorganic precursors of the second phase. The method is illustrated into two case studies; the former, on Zirconia Toughened Alumina (ZTA) materials for orthopedic applications, and the latter, on Zirconia-based composites for dental implants, discussing the advances and the potential of the method, which can become a valuable alternative to the current synthesis process already used at a clinical and industrial scale.

## 1. Introduction: A Brief History from Pure Monolithic to Composite Bioceramics

The use of ceramics has been growing in the past 40 years in the biomedical field. Their main applications include orthopedic and dental devices, being mainly applied as replacements for hip, knee, teeth, and as bone gaps filler. Depending on their *in vivo* behavior, they are classified into bioinert, bioactive, and/or bioresorbable [1]. In the first case, as a rule, the material has a high chemical stability *in vivo*, as well as a high mechanical strength, and, when incorporated in a living bone, it shows a minimum interaction with the surrounding tissue. On the other hand, bioactive ceramics present the capability of inducing a favorable response from the host tissues (e.g., inducing strong chemical bonds with bone and favoring bone ingrowth), but their mechanical properties are generally lower than those of bioinert materials. Finally, bioresorbable ceramics have the unique ability to degrade gradually, being replaced by the natural tissue.

This wide spectrum of biological interactions leads to a broad range of engineering design and strategies. In fact, bioactive materials (such as hydroxyapatite and tricalcium phosphate) have poor mechanical properties, so their applicability is confined to implants that do not have to bear significant loadings, and the main requirement is to provide favorable surfaces for biological bonding and bone ingrowth. Otherwise, the critical conditions in joint replacement restrict the choice of materials to the harder and stronger ones, such as alumina (Al_2_O_3_) and zirconia (ZrO_2_), even though they are not able to create (at least at the moment) a bone-implant interface and cannot be used as bone filler [1].

According to the previous classification, this review focuses on bioinert oxide ceramics, and, most of all, on alumina and zirconia-based materials for load-bearing applications, such as hip and knee bearings and dental devices. These ceramics present drastically reduced wear rates and excellent long-term biocompatibility, which can increase the longevity of the prosthetic joints. This benefit is clinically important, as hip and knee replacement has become a very common surgical procedure, being performed in increasingly young and active patients. The main characteristics for materials to be used in total hip arthroplasty (THA) are [2]:

(i) High mechanical properties (in terms of flexural strength, elastic modulus, fracture toughness, and fatigue resistance). It should be kept in mind that the loads in the body can vary from three times the body weight (~3 kN) for normal walking to eight times the body weight (~8 kN) for jogging or stumbling;
(ii) High corrosion resistance and biocompatibility *in vivo*;
(iii) High hardness and good surface finish, for ensuring long term wear resistance;
(iv) Good wetting, for providing good lubrication between the implant surface and the synovial fluids.

According to such properties, Al_2_O_3_ was introduced as a candidate material for orthopedic bearings in the 1970s, whereas partially stabilized-ZrO_2_ appeared in the mid-1980s.

Alumina is, by far, the most widely used ceramic in THA. However, the early clinical applications showed a high fracture rate (~13%) of alumina devices, later imputed to an incomplete densification of the material [3]. In order to improve, as much as possible, the mechanical properties of alumina, efforts addressed the use of high purity raw powders and the application of unconventional sintering techniques, such as hot isostatic pressing (HIP), for reaching full densification. Such advances gradually increased the sintered density and decreased the alumina average grain size, giving rise to the last generation of alumina ceramic components, characterized by excellent mechanical properties (flexural strength higher than 550 MPa, Vickers hardness of 1800–2000 HV and a low fracture rate of about 0.004%–0.015%) [3,4]. Technical data of first, second, and last-generation polycrystalline alumina are reported in Table 1 [2], showing that the third-generation materials completely fulfill the International Standard Organization (ISO) requirements for medical-grade alumina implants [1,4], reported in the same Table.

**Table 1:** Properties of various medical-grade Alumina ceramics and International Standard Organization (ISO) requirements [1,2,4].

Property	Alumina: 1970s	Alumina: 1980s	Alumina: 1990s	ISO Alumina Standard 6474
Density (g/cm^3^)	3.94	3.96	3.98	>3.90
Mean grain size (μm)	4.5	3.2	1.8	<7
Bending strength (MPa)	400	500	580	>400 (in Ringer’s solution)
Vickers Hardness (HV)	1800	1900	2000	>2000
Hot Isostatic pressed	No	No	Yes	–

Despite the mentioned good properties, there are two major concerns for the reliability of alumina bearings: the low fracture toughness (~4 MPa·m^1/2^) and the susceptibility of alumina to failure through slow crack growth at stresses well below to the ultimate fracture strength. The explanation of this last behavior will be given later. Alumina is, therefore, restricted to standard designs (for example ball heads with a diameter larger than 28 mm), for which the risk of failure is now exceptionally low. The development of new concepts or products will require new ceramics with better intrinsic crack resistance. Alumina has been also used for dental devices, starting from Dr. Sami Sandhaus, a Swiss dentist, who developed a medical device entirely made of polycrystalline alumina, the CBS^®^ dental implant [5]. This dental device enjoyed good clinical success, thus leading to the development of other highly pure alumina implants. In addition to the advantages of aesthetic and biocompatibility, alumina dental implants have shown several shortcomings in terms of design, stiffness, and modest fracture toughness, similarly to what already has been observed for THA. The low flexural strength and the flaws introduced during the surface grinding have caused failures on these devices leading to their demise in favor of titanium dental fixtures [5]. 

**Figure 1:**
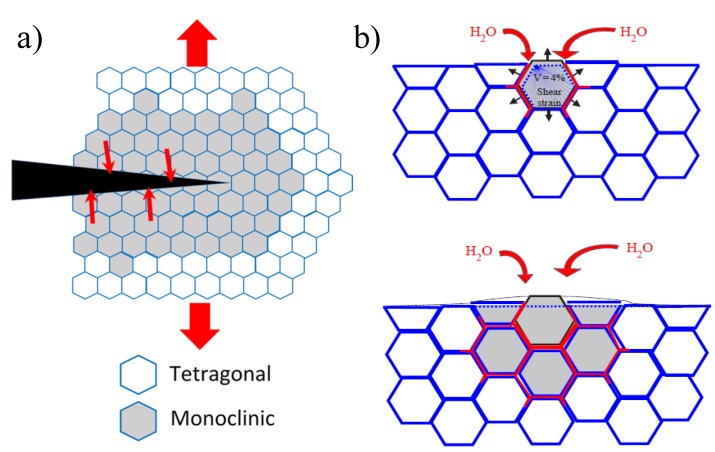
Schematic illustration of (**a**) phase transformation toughening and (**b**) ageing [16]. In (**b**): Nucleation on a particular grain at the surface, leading to micro-cracking and stresses to the neighbors (**top**); growth of the transformed zone, leading to extensive micro-cracking and surface roughening (**bottom**). Red path represents the penetration of water due to micro-cracking around the transformed grains.

Zirconia femoral heads were introduced in the 1980s in response to the brittleness of alumina and the consequent potential failure of implants [6,7]. Biomedical grade zirconia exhibits the best mechanical properties of oxide ceramics, well superior of those of alumina. In fact, the zirconia fracture toughness is almost the double of that of alumina (6–8 MPa·m^1/2^ *versus* 3–4 MPa·m^1/2^) and the strength can reach values around 2 GPa for a fine-grained material [2]. This exceptional mechanical strength is due to the phase transformation toughening mechanism, able to increase the crack propagation resistance in zirconia. To explain such phenomenon, we should introduce the three polymorphic zirconia forms, being progressively the monoclinic (*m*), tetragonal (*t*), and cubic (*c*) phases as temperature increases. By adding suitable stabilizers, such as yttrium oxide (Y_2_O_3_), the *t* phase can be retained at room temperature in a metastable phase, being able to re-transform into the stable *m* phase under an applied stress. This martensitic transformation (*t* to *m* phase) involves a volume expansion of ~3%–5% and large shear strains (~7%) [8] able to induce compressive stresses at the crack tip that avoid the crack propagation, leading to the well-known toughening effect. A scheme of the transformation toughening mechanism is given in Figure 1a [9].

The mostly used biomedical grade zirconia typically contains 3 mol% yttria (3Y-TZP). The sintered material commonly consists of a single phase (the metastable *t* phase), fine-grained (0.1–1 μm), fully densified microstructure.

Despite the excellent properties of zirconia ceramics, some compositions, such as 3Y-TZP, present a major drawback in moist atmosphere, since they undergo Low-Temperature Degradation [9,10,11], as shown in Figure 1b. This is an ageing phenomenon that causes loss of strength and generation of micro-cracking in presence of water. It consists of a slow transformation of metastable *t* zirconia to the *m* phase (without any applied stress) in an important temperature range, typically from room temperature up to around 400 °C, thus including the temperature used for steam sterilization (~140 °C) and the human body temperature (37 °C) [9,10]. Although Low Temperature Degradation has been studied for more than 20 years, the precise mechanism by which moisture catalyzes the phase transformation is still not fully clarified [9,10]. Several experimental results show that moisture, in the form of OH^−^ ions, diffuses into the zirconia lattice during exposure to humid atmosphere. Most probably, the oxygen of environmental water is located on vacancy sites, whereas the hydrogen is placed on adjacent interstitial sites. This highlights the role of oxygen vacancies initially present in zirconia on water diffusion rate. This also emphasizes the effect of stabilizers inside the zirconia lattice: in Y-TZP, many oxygen vacancies are generated by the trivalent cation (Y^3+^), making the water diffusion rate higher than in other zirconia-based ceramics stabilized with tetravalent cations, such as CeO_2_-doped ZrO_2_ (Ce-TZP), in which Ce^4+^ does not induce vacancies [12]. Schubert and Frey [13] showed that the penetration of OH^−^ leads to a lattice contraction, inducing tensile stresses in the surface of the grains. This destabilizes the *t* phase and favors its martensitic transformation to the *m* phase. The mechanism proceeds then in an autocatalytic way, since the transformation of one grain leads to a volume increase, stressing up the neighboring grains, and leading to micro-cracking. This offers a path to water to penetrate inside the sample [12].

Low Temperature Degradation has been known for more than 20 years [14], but its effects were underestimated at ambient temperature until the end of the 1990s, when Chevalier *et al.* showed that it could proceed under *in vivo* conditions with time scales in the order of only some years [15]. In 1997, the US Food and Drug Administration (FDA) reported on the critical effects of standard steam sterilization procedure (134 °C, 2 bar pressure) on the surface roughness of zirconia implants [16,17]. In 2001, the Therapeutic Goods Administration in Australia announced a large series of failures (more than 800) in batches of implants processed with a new furnace technology by Saint-Gobain Desmarquest Prozyr^®^ [16,17]. This event had a catastrophic impact for the use of zirconia, and the market sale decreased of more than 90% between 2001 and 2002, with no evidence of clear renew.

Quite surprisingly, in the same years the dental community discovered the use of zirconia [18,19,20] and, apparently, without concerns about aging [11,21,22]. Inlays, onlays, single crowns, and fixed partial dentures have been realized by using zirconia core. Moreover, also implant abutment and implants are today available in zirconia [7]. Such a wide application explains the continuous increase (of more than 12% per year) of the zirconia market in the dental field [16], the success being imputable to the zirconia esthetic properties, in addition to its mechanical specifications. Once again, Y-TZP ceramics exhibits the best combination of mechanical and esthetical properties among polycrystalline oxide ceramics. 3Y-TZP materials with a translucently of 12%–15% are available, and the color can be adjusted by doping, for example, with iron or rare earth elements, meeting the demand of natural-like restoration [17]. If ageing has been well documented in the orthopedic field, the lack of studies for dental applications is striking. Even though a few general papers devoted to dental zirconia underline the need to “keep in mind that some forms of zirconia are susceptible to ageing and that processing conditions can play a critical role on the Low Temperature Degradation of zirconia” [23] the problem of ageing in dental zirconia is still underestimated. In part, this is due to the availability of new ageing resistant 3Y-TZP ceramics (such as TZ3Y-E developed by Tosoh), but also to a lack of exchanges from one community to another. The ageing consequences may seem less important in dental applications, especially when restorations are concerned, but if large-scale failure events such as those of Prozyr^®^ heads occur, it would be a critical issue for zirconia and for ceramics in general [17].

In order to overcome the low toughness of alumina and the sensitivity to ageing of zirconia, the trend today is to develop alumina-zirconia composites. This can be the way to get benefits from the zirconia transformation toughening mechanism without the major drawback associated with its transformation under steam or body fluid conditions. A recent literature overview on alumina-zirconia composites developed for biomedical applications show that different compositions, from the zirconia rich side, to the alumina rich one have been tested.

Concerning the alumina-rich compositions (alumina in the range 60–95 vol%), Zirconia Toughened Alumina (ZTA) composites have been widely investigated. Starting from June 2000, ZTA became available as femoral head material, commercialized by CeramTec AG (Plochingen, Germany) under the trade name of BIOLOX^®^ delta. After approval by FDA in 2003, ZTA started to be widely used in THA: in the last 10 years, approximately one million CeramTec ZTA femoral heads and over 700,000 inserts have been implanted worldwide. A second ZTA product, AZ209, was introduced in the Japan market by KYOCERA Medical (Osaka, Japan) during 2011 [24]. 

In ZTA, the zirconia particles can exhibit a *m*-phase or a *t*-phase, but the higher mechanical properties (strength and fracture toughness) have been observed in the latter case. A critical factor is, however, the ZrO_2_ grains size, which should be optimized by controlling the composition and processing conditions. In fact, the zirconia grain size must range between two critical values, the *D_c_*′ below which the transformation is completely hindered (even under stresses at the crack tip), and the tetragonal phase is stabilized by its very small size, and the *D_c_* above which spontaneous transformation occurs during cooling the composite system from the sintering temperature [25]. These critical sizes depend on the zirconia amount into the ZTA composites and on the oxide stabilizer nature and content: for instance, values in the range 0.6–2.0 μm for the upper limit, and 0.1–0.4 μm for the lower one have reported in literature for ZTA containing 8–15 vol% of un-stabilized ZrO_2_ [26].

Because of the *t*-particles are present within the alumina matrix, the strength degradation by hydrothermal ageing in ZTA is limited, as compared to monolithic Y-TZP. However, remembering that ageing occurs by nucleation and growth mechanisms, starting at the surface, the Y-TZP or zirconia particles should not form a continuous network in the alumina matrix, meaning that their content should be below the percolation limit (~16 vol%) [2,27].

When un-stabilized zirconia particles are added to the alumina matrix, the metastabilization of the *t* zirconia grains is achieved thanks to their very fine grain size and to the presence of the stiffer alumina phase. Therefore, if the addition of yttria is avoided, the formation of oxygen vacancies inside the zirconia lattice is reduced, thus, limiting the diffusion of water radicals in the zirconia ceramics [15].

Hip simulator tests indicate that the wear of ZTA-on-ZTA is lower than that of Alumina-on-Alumina. The ZTA/ZTA couple displayed the highest resistance to wear and the lowest amount of surface roughness after five million cycles [24]. However, ageing of ZTA containing 14 vol% Y-TZP particles was observed when tested for long time (19 months) in Ringer’s solution, promoting the formation of a *m*-ZrO_2_ surface layer which leads to a significant reduction of the flexural strength [2].

These results demonstrate that, in spite of the advances shown by ZTA composites with respect to monolithic alumina and zirconia materials, there is still room for further optimization, in terms of both composition and architecture of the composites.

An example is given by Biolox delta^®^ manufactured by CeramTec AG (Plochingen, Germany). This company produces a ZTA material, which also contains small amounts of SrO and Cr_2_O_3_ [28]. During sintering, these additives react with alumina, leading to the *in situ* formation of plate-like aluminates. This approach, which is becoming increasingly popular [29,30,31,32], brings to a fracture toughness increment, since the elongated grains (with high aspect ratios) can induce additional toughening mechanisms by crack deflection and crack bridging [33]. By optimizing the microstructure and the sintering conditions, materials with remarkable mechanical properties are produced, being the flexural strength higher than 1200 MPa, the fracture toughness of 6.5 MPa·m^1/2^ and the Vickers hardness of 1975 HV [2].

In the zirconia-rich side, alumina-toughened zirconia (ATZ) composites are currently produced. The addition of Al_2_O_3_ to Y-TZP up to 0.25%, allows increasing the bending strength from 1100 to 1200 MPa and improving the resistance to ageing [20]. However, when the alumina content was increased to 25% and the composite was sintered by HIP, the highest strength—over 2000 MPa, was yielded. ZIRALDENT^®^, manufactured by Metoxit AG and having the above composition, is at the present the strongest biomedical ceramic known.

**Figure 2:**
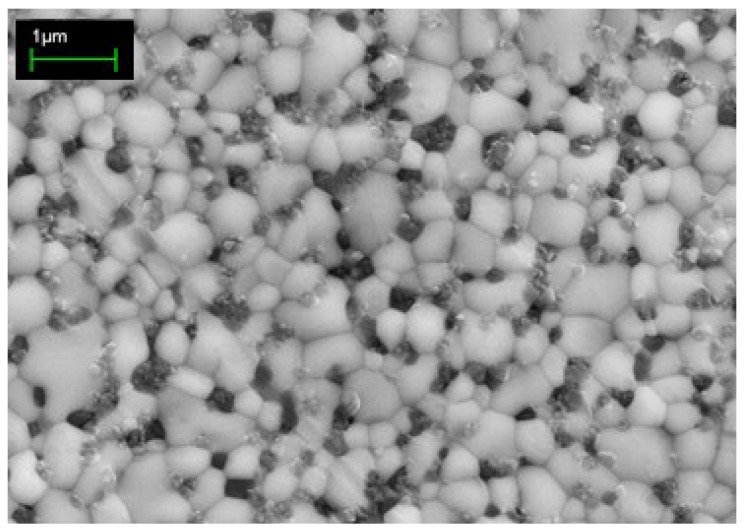
Sintered microstructure of 10 Ce-TZP/16 vol% MgAl_2_O_4_ composite [38].

In the case of Ce-TZP/Al_2_O_3_ composites, the first investigation belong to Sato in 1989 [34], proving an increase of the hardness and of the Young’s modulus of this material with respect to pure Ce-TZP ceramics. However, the best results were reached by Nawa [35,36], in 1997, when he developed an intra-granular microstructure, in which several nano-sized (10–100 nm) Al_2_O_3_ particles were trapped into zirconia grains. Nawa observed that the hardness, the elastic modulus and the fracture strength increased, while increasing the alumina content. At the same time, he observed a decrease of the fracture toughness, in agreement with the decrease of transformability. Thanks to the precise control of the microstructure and composition, the mechanical properties were optimized (bending strength and fracture toughness of 1290 MPa and 8.62 MPa·m^1/2^, respectively), giving rise to the commercial product named NANOZR^®^ (Panasonic Electric Works, Tokyo, Japan [5,37]). More recently, a new type of zirconia matrix composite based on Ce-TZP/magnesium spinel (MgAl_2_O_4_) system was developed [38] (Figure 2), showing a good combination of high strength (about 900 MPa) and toughness (15 MPa·m^1/2^).

In addition, for this class of composites, elongated grains inside the zirconia matrix have been already used: Ce-TZP composites reinforced by LaNbO_4_ [39], by strontium [40], barium [41], and lanthanum [42,43] hexaaluminate were previously reported in literature. More complex quasi-ternary aluminates, such as BaMnAl_11_O_18_ and CeMnAl_11_O_19_, were investigated as well [44,45]. In spite of such publications, the rule of the elongated grains inside a zirconia matrix is still unclear: they have been supposed to provide crack bridging and deflection effects, but these mechanisms are not yet clearly demonstrated.

What here described highlights that the major drawbacks of pioneering pure monolithic bioceramics can be overcome by the use of composites. However, the ability to tailor the microstructure of ceramic composites is essential in order to fulfill the requirements of biomedical materials: for this reason, the following paragraph reports and briefly discusses the microstructure-properties relationships, already observed in ceramics used in structural biomedical applications.

## 2. Microstructure-Properties Relationship in Composite Bioceramics

In order to better understand the role of the microstructure, we should recall that failure of ceramics *in vivo* commonly results from slow crack growth under static or repetitive loading experienced in the body, until fracture. As extensively described in literature (see for example [2,46]), this phenomenon can be understood as a corrosion-assisted crack propagation process, leading to a progressive loss of strength. In fact, under an applied tensile stress σ, the stress at the crack tip can be described as the stress intensity *K*_I_ given by:

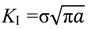
(1)
where 2*a* is the length of the crack. It is generally assumed that fast failure occurs in brittle materials if *K*_I_ becomes equal to (or greater than) the critical stress intensity factor, *K*_IC_, commonly known as fracture toughness. The fracture strength of a brittle material can be written as:

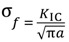
(1)


According to Equation (2), it is evident that the fracture strength is determined by the fracture toughness and the flaw size. This equation represents a critical value for *fast crack growth*. Additionally, it is now well recognized that brittle materials can be also susceptible to *slow crack growth* (SCG, also referred to as *subcritical crack growth*), meaning that they are sensitive to the applied stress, as well as to environmental factors, such as water, water vapor, and temperature. The combined effect of high stress at the crack tip and reaction with water (or body fluids) allows the crack to grow, until reaching the critical length for failure at the stress level (Equation (2)), where the material fails. Thus, many efforts have been devoted to determine a threshold in stress intensity factor (*K*_I0_), corresponding to the condition of zero crack velocity, at which crack propagation does not occur. Being *K*_I0_ the safe range for using ceramics in THA, this parameter has been determined for both monolithic and composite bioceramics [46].

**Figure 3:**
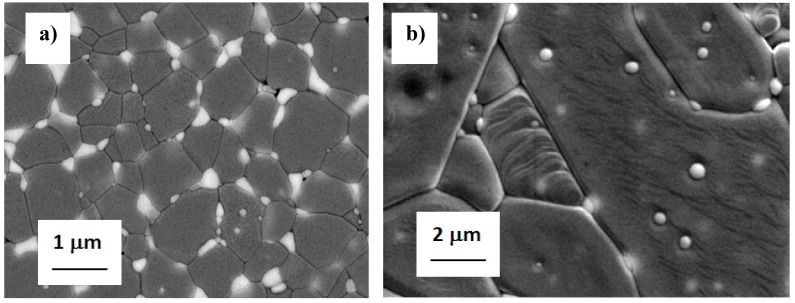
Sintered microstructures of (**a**) micro-; and (**b**) nano-ZTA composites [46,47].

**Figure 4:**
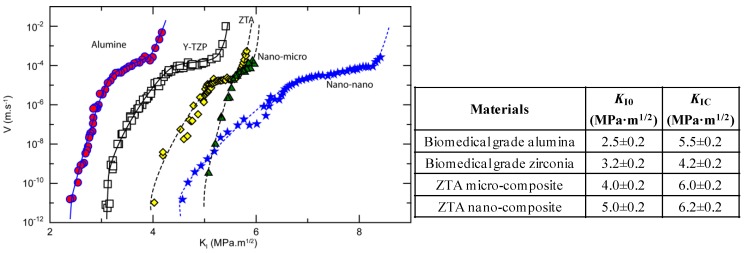
Crack velocity (*V*) versus stress intensity factor (*K*_I_) for biomedical grade alumina, yttria stabilized zirconia, ZTA micro-composite, and ZTA nano-composite. Enclosed to the Figure, the values of *K*_I0_ and *K*_IC_ for the same materials [46,47].

Chevalier *et al.* [46] clearly demonstrated a higher threshold for subcritical crack propagation (*K*_I0_) for Alumina-10 vol% ZrO_2_ composite, as compared to both pure alumina and 3Y-TZP materials, indicating a higher reliability and expected lifetime for the composite. More interestingly, the same authors [47] compared the SCG propagation behavior of the previous ZTA micro-composite with that of a nano-composite containing only 1.7 vol% of nano-sized ZrO_2_ grains (average size of about 150 nm) embedded in a micron-sized alumina matrix (average size of about 5 μm) [47], where they predominantly occupied transgranular positions. The microstructure of the ZTA micro- and nano-composites are shown in Figure 3, whereas the SCG behavior of the monolithic and composite materials is depicted in Figure 4, determined by the crack velocity (*V*) *versus* the stress intensity factor (*K*_I_).

From this figure, it is possible to estimate the threshold (*K*_I0_) for subcritical crack propagation, determined from the points on the *V-K*_I_ diagram below which there is an abrupt drop of the crack velocity (V < 10^−12^ m· s^−1^). On the other hand, the toughness (*K*_IC_) was determined by extrapolation of the *V-K*_I_ curve to high crack velocities (10^−2^ m· s^−1^). These values, for the different ceramics, are given in the Table within the Figure 4. 

From the above figure, we can see that zirconia exhibits a higher *K*_I*C*_ than alumina, but that their thresholds are quite close, meaning that the necessary crack tip stress to initiate slow crack growth is quite similar in both materials. The higher toughness of zirconia can be attributed to its transformation toughening effect; on the other side, zirconia bonds are prone to chemisorption of the polar water molecules, more than the alumina ones, explaining the lower slope of the *V-K*_I_ curve of zirconia as compared to that of alumina. The ZTA micro-composite has both larger *K*_IC_ and *K*_I0_ values than the two monolithic ceramics. In fact, thanks to the alumina matrix, this composite shows a low susceptibility to stress assisted corrosion by water (or body fluid). However, as it is reinforced by transformable zirconia particles, its *V-K*_I_ curve slop is similar of that of alumina, but shifted towards higher *K*_I_ values. Finally, we can see that the ZTA nano-composite is characterized by similar *K*_IC_ as the micro-composite, but a significantly higher *K*_I0_ value. In the nanocomposite, only a small fraction of nanosized ZrO_2_ grains into the alumina matrix is needed to dramatically increase its crack resistance, even if the transformation toughening mechanism has to be ruled out since the *t* grains transformation is hindered by their small size. The behavior of the ZTA nanocomposites was instead attributed to the residual compressive stress field inside the alumina grains, generated by the transgranular zirconia particles, and having a strong impact on the SCG resistance.

Hydrothermal ageing also critically depends on the microstructure, and, hence, on the processing. A key point is producing a fully dense microstructure and, more important, free from percolative porosity [17]. We recall that the failure of Prozyr^®^ femoral heads, *i.e.*, the spontaneous disintegration because of accelerated ageing, occurred in batches processed in a new tunnel furnace causing a lack of densification at the center of the head [17]. For the same reason, the current industrial production of ceramic femoral heads implies two sintering steps to assure full densification: pressureless sintering followed by post hot isostatic pressing, being the first step unable to guarantee to the bodies the end product specifications (see Table 1).

When zirconia grains are stabilized by yttria, the control of the size and distribution inside the ZTA composite is of primary importance. It has been shown that, if the zirconia amount is above the percolation limit (~16 vol%), the composite might be sensitive to aging. Below this critical amount, only the largest zirconia grains (or the zirconia clusters eventually present in non-homogeneous materials) can undergo ageing, the smaller ones being over-stabilized by the stiffer alumina matrix. In order to deepen the role of zirconia aggregates on the Low Temperature Degradation behavior, Gutknecht *et al.* [48] investigated the ageing sensitivity of two batches of ZTA composites (containing 13 vol% of 3Y-TZP) both processed by the powder mixing method, but using different polyelectrolyte dispersants. Under optimal electrostatic dispersion [48], the composite was completely free from aggregates and did not exhibit any ageing phenomena. On the opposite, when a not appropriate dispersant was used [49], the final microstructure presented a severe aggregation of zirconia grains and ageing was observed especially during the first two hours of autoclave treatment. In agreement with this result, a colloidal method was used to produce a ZTA composite [47], made by zirconia nano-particles uniformly distributed in the alumina matrix, which was almost ageing-free.

Many other specifications of biomedical grade ceramics depend on their microstructure. For instance, to gain benefit from the transformation toughening mechanism, the zirconia particle size should range between two critical values as previously mentioned: the highest relates to the size for spontaneous transformation of the *t*-phase to the *m*-phase during cooling, and the lowest to the size for which no transformation to the *m*-phase is possible. Both critical sizes depend on the stiffness of the matrix and on the amount and composition of the zirconia particles. Generally, zirconia particle sizes of a few hundred are observed [17]. More in general, the toughness-strength relationship depends on the microstructure, including the amount and type of zirconia, grain size of matrix and second phase, location of the zirconia grains inside the matrix [46,50,51,52,53].

Finally, also the optical properties of the ceramics depend on the microstructure: translucency of technical ceramics may be achieved with a very fine grain size (submicron) and low porosity content (lower than 1% or even 0.1%). An example is given by the fully dense translucent Y-TZP ceramics when processed with a grain size lower than 0.5 μm, meeting the demand for both natural-teeth-looking restoration and high mechanical strength [12].

## 3. Synthesis Methods to Produce Biomedical-Grade Ceramic Composites: A Focus on the Surface-Coating Strategy

Zirconia- and alumina-based composite powders have been produced by several elaboration techniques, from the traditional milling–mixing [48,49,54] to wet chemical routes (such as alkoxide-based route [55], sol-gel [56,57,58,59,60], co-precipitation [61,62], and hydrothermal methods [63]).

As stated in literature, milling-mixing procedures are the most applied method, in which the powders are generally mixed in form of oxides to produce bi- and tri-phasic composite materials [35,38,40,54,64,65,66]. When powder mixing is performed, it is mandatory to check the characteristic of the starting powders and to carefully control the dispersion and stabilization degree of the mixed suspension.

Concerning the characteristics of the powder, size and size distribution, shape and morphology, agglomeration degree, phase composition and surface properties have to be considered. In fact, a wide size distribution on one hand leads to a higher packing density in the green bodies. On the other hand, the precise control of the microstructural development during sintering could be difficult to achieve because the larger grains can grow at the expense of the smaller ones. At the same time, the particle size influences the final grain size and the densification rate. Due to the higher specific surface area, the densification rate increases as the particle size decreases [67]. In addition, if the powder is agglomerated, the particle packing in the green body will be heterogeneous, giving rise to differential sintering rates and to heterogeneous microstructures [68]. 

When wet-forming methods are used, the characteristics of the mixed suspension have to be carefully controlled. In fact, it is well recognized that microstructural defects, aggregates or irregular phase distribution of the second phase in the ceramic matrix can arise from a non-optimized or unstable mixed suspension [49]. Thus, the dispersion degree (and eventually the nature of dispersant selected), the homogeneity and the rheological properties of the suspension have to be investigated.

Furthermore, when the powder mixture contains a relevant fraction of nanocrystalline particles, additional complications can arise. The use of high shear forces during dispersion is sometimes necessary to homogeneously distribute the nanoscale fraction into the liquid. Separation by selective agglomeration or sedimentation during the shaping process can also occur, thus, affecting the final microstructure. Finally, when prolonged milling is carried out, contamination of the powder from jars and milling spheres could occur.

In order to overcome such drawbacks, several wet chemical routes were exploited in the last few years. Among them, sol-gel and co-precipitation are the mostly employed.

In the sol-gel method, inorganic salts or metal-organic compounds are used for the sol preparation. Then, by hydrolysis and condensation reactions, the sol is converted into a gel that has to be dried, to eliminate the exceeding liquid. Advantages of this technique include the precise control of the particles morphology and size, the relatively low processing and crystallization temperatures of the final phases, the product homogeneity, the chemical purity and the full control of stoichiometry. However, the complexity of the method increases when multi-cation materials (such as in the alumina-zirconia system) have to be prepared. In fact, the hydrolysis and condensation reactions have to be even more carefully controlled (solution pH, temperature, and reactant concentration), in order to avoid selective segregation of one, or more, metal ions. Concerning the other disadvantages of wet-chemical methods, it is known that the chemical precursors are expensive and often sensitive to moisture; the dried product is often hard agglomerated and sometimes it is difficult to preserve the characteristics of a lab-scale production when large batches of powders are concerned [69].

In the precipitation technique, the solubility of the metal ions dissolved in aqueous solution is exceeded by evaporation of the liquid or by adding a chemical reactant. Thus, the precipitation of metal hydroxides is promoted. The modification of the pH and temperature of the solution allows controlling nucleation and growth mechanisms and consequently the particles morphology [70,71]. In addition, in the case of co-precipitation, it is necessary to achieve the suitable conditions for the simultaneous and quantitative precipitation of all the species involved [72].

An example of the role of various wet-chemical synthesis methods on the final microstructure of Al_2_O_3_-ZrO_2_ composites is given in the work of Rana [62]. Here, three different processing routes were employed to prepare composite powders, namely gel precipitation (GPT), precipitation (PPT), and washed precipitation (WPT). In all three cases, starting raw materials were zirconium and aluminum chloride and aqueous ammonia was added to induce precipitation. During the synthesis, the pH was maintained in the range 6–6.5 (gelation point) for GPT, whereas for the other two routes precipitation was carried out at a higher pH, in the range 8.7–9.1. The difference between the PPT and WPT routes was the washing process: in the former case, the precipitate was separated from the liquid and then dried; in the latter, it was washed with hot water and alcohol before drying. These different processing routes affected the crystallization temperature of the amorphous powder, as well as the phase evolution of Al_2_O_3_ and ZrO_2_ phases during calcination. In fact, while GPT and PPT powders crystallized at 350 °C, no crystallization was observed in WPT powder until 650 °C, this latter being the only product able to produce pure *t*-phase. The powder agglomerates size was largest for GPT than for WPT. Moreover, GTP route produced hard agglomerates, while low agglomeration strength was observed in WPT powders. As expected, soft agglomerated powders gave rise to compacts with good green density and able to sinter to a higher density at lower sintering temperatures as compared to GPT and PPT products.

Hydrothermal oxidation is an alternative wet-chemical method to prepare alumina-zirconia composites powders. An example is provided by the work of Chen and Chiao [73], where ZrA1_3_ and Zr_5_A1_3_ alloys have been used as starting materials. It was found that the sizes of the aluminum and zirconium oxides were lower than those of the respective powders obtained by the hydrothermal oxidation of aluminum and zirconium metals separately, this reduction of size being attributed to the retarding effect of the alumina particles on the growth of the zirconia particles.

In the last years, innovative procedures consisting in the surface modification of a commercial powder with the second phase precursors have been exploited. They can be considered as a compromise between the powder mixing technique and the colloidal ones, allowing a deeper control of the final microstructure. In fact, the close mixing between the matrix ceramic nanoparticles and the metal ions, precursors of the second phases, is realized at the atomic/nano level, assuring an excellent distribution of the second phases in the composite powders. On the opposite, in the case of milling methods, the second phases are in the form of a solid precipitates (such hydroxides) or solid particles, leading to a less effective mixing with the matrix powder.

Schehl *et al.* [55] developed a modified colloidal method for the production of alumina composites. Briefly, this method consists in grafting alumina commercial powders by using organic precursors (metal-alkoxides) of the second phases. Thanks to the use of organic media (typically ethanol), the addition of metal alkoxides initiates a substitution reaction between the metal alkoxide and the OH groups located on the particles surface. After drying and calcination, second-phase nanoparticles are formed *in situ* on the alumina particles surface. This method was applied to various alumina-based composite materials, with Zirconia, Yttrium Aluminum Garnet (YAG) or mullite second phases, showing in all cases a fine and homogeneous microstructure. The mechanical properties of the ZTA composites obtained by this route were compared to those of the same materials processed by classical mechanical mixing. The colloidal-route processed composites showed finer microstructures and better mechanical properties, particularly a higher *K*_I0_ value [46] and a slower degradation rate [49].

Beside the method investigated by Schehl *et al.* [55] based on the use of organic precursors, also inorganic salts were used to coat the surface of commercial alumina particles and to induce the crystallization of second phases upon calcination. Yuan *et al.* [74], for instance, added cerium and aluminum nitrates to the isopropanol suspension of zirconia powder, in order to obtain 12Ce-TZP/2 wt% Al_2_O_3_ composites. The as-obtained suspension was mixed for 48-h on a multidirectional mixer and then water and alcohol liquid media were removed by means of a rotating evaporator at 95 °C, thus promoting the formation of the desired final phases. The dried powder was subsequently calcined in air at 800 °C for 1 h in order to obtain the Al_2_O_3_-doped CeO_2_-coated ZrO_2_ nanopowder.

More recently, we developed an alternative method to produce alumina- and zirconia-based composites, inspired on the concept of “surface modification” of commercial oxide powders [75,76,77,78], which shows some progress with respect to the previous described techniques. In fact, in our process, only inorganic precursors and aqueous media are used, making this strategy much more simple and potentially transferable to a pre-industrial scale production. A second difference lays in the mixture drying method, which we perform by means of a “flash” drying technique, such as atomization, in which the liquid medium is converted into fine droplets and instantaneously evaporated. This step has a key role in the process, since the homogeneity of the mixture is “frozen” in the dried products, completely avoiding the segregation of the metallic dopants, as can occur by slow drying in an oven. The method was successfully applied to alumina-based bi- and tri-phasic composites and, more recently, successfully exploited for the elaboration of zirconia-based composites with complex compositions and microstructures, containing both equiaxial and elongated second phases.

In order to show the potential of this new technique, in the following we present two case studies, the former devoted to alumina-zirconia composites to be potentially used for orthopedic applications, the latter focusing on zirconia-based tri-phase composites for dental industry. The process is being developed in the frame of the European Project “*Longlife*” (FP7, grant agreement No. 280741) and recently registered as an Italian Patent (TO2014A000145, registered on 21 February 2014).

## 4. Case Studies

### 4.1. ZTA for Orthopedic Applications

The surface modification route was used to prepare ZTA composites, in which the zirconia content ranged between 5 and 20 vol%. The starting raw material was a commercial α-alumina powder (TM-DAR, supplied by TAIMEI Chemicals Co., Tokyo, Japan) characterized by nanosized primary particles (average size of about 100 nm [79]) but presenting a certain agglomeration degree, since by laser granulometry we determined agglomerates of about 30 μm in size [76]. Zirconium (IV) chloride (ZrCl_4_, >99.5% purity, supplied by Sigma-Aldrich) was selected as zirconia precursor. Further details on the elaboration method can be found elsewhere [75,80,81].

In this process, a key point to obtain a homogeneous distribution of the zirconia second phase in the alumina matrix is, first of all, to achieve a close and homogeneous mixing between the matrix powder and the zirconium ions contained in the starting aqueous suspension. This can be obtained on one side by reducing the size, or eliminating, the alumina agglomerates from the starting powder, and on the other side by avoiding the formation of second-phase precipitates, such as Zr(OH)_4_.

For this reason, the starting alumina powder was first dispersed alone. Aqueous suspensions at 50 wt% solid loading were ball-milled using α-Al_2_O_3_ milling spheres, the powder/sphere ratio being 1/5. Few drops of dilute hydrochloric acid were added to the suspension, lowering the slurry pH from the starting value (about 6.5) to 4.5, far from the isoelectric point of alumina [82]. In this way, a good de-agglomeration degree was achieved after 3 h of ball milling, reaching an average particle size of about 0.17 μm [83]. A zirconium chloride aqueous solution was then drop-wise added to the well-dispersed alumina suspension. Since Zr^4+^ ions show a strong hydrolysis, when ZrCl_4_ was dissolved in distilled water and added to the alumina slurry, the suspension pH decreased to <1. To increase the pH to acceptable values, tribasic ammonium citrate acting as a chelating agent for Zr^4+^ was also added. In this way, it was possible to maintain the suspension pH to about 4.5, without inducing the precipitation of Zr(OH)_4_ [84].

The mixed suspension was spray-dried and the product was submitted to various thermal pre-treatments, in the range 400–1000 °C, in order to decompose the synthesis by-products (mainly, ammonium chloride) and to induce the crystallization of zirconia grains onto the surface of the alumina particles.

The precise follow up of the zirconia crystallization at the surface of alpha-alumina grains during the thermal pre-treatments was deeply described in a previous paper [85]. We report here some results, with the aim of showing the potential of the surface coating route in tailoring the size and distribution of the ZrO_2_ nanocrystals on the surface of alumina particles. In fact, as the process involves the *in situ* crystallization of the ZrO_2_ grains, by carefully controlling the thermal treatments it is possible to control the crystalline fraction, the crystallites size, the kinetics, and mechanisms of nucleation and growth.

**Figure 5:**
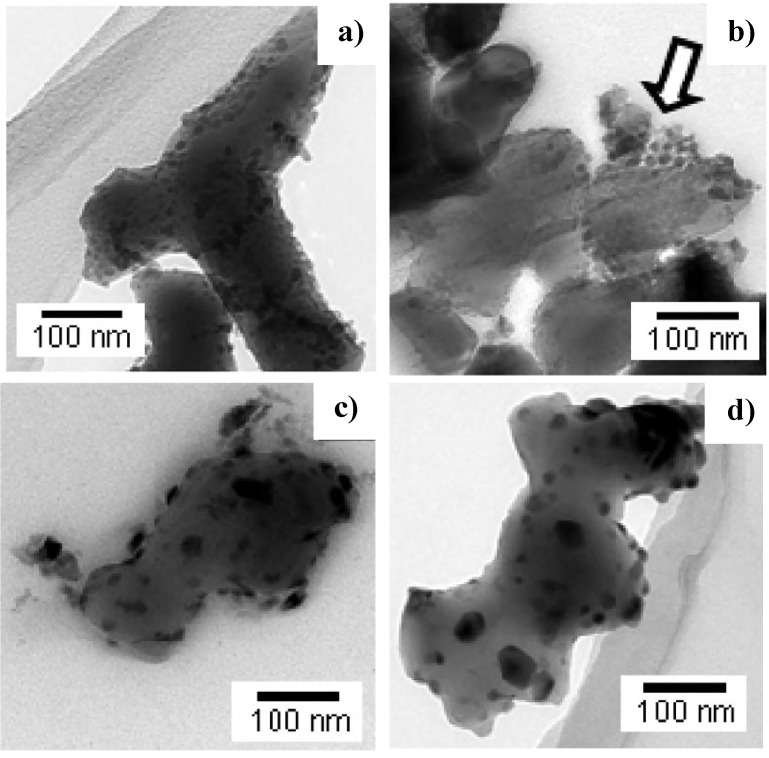
TEM images of ZTA powder after treatments at different temperatures and times: (**a**) 600 °C–1 h; (**b**) 600 °C–10 h; (**c**) 1000 °C–1 h; (**d**) 1000 °C–10 h. The arrow indicates the zirconia nuclei dragged into discrete pockets [83].

By TEM observations, it was found that powders treated up to 500 °C are composed by alumina particles surrounded by an amorphous layer, which progressively disappeared by increasing the calcination temperature. At the same time, the zirconia crystallites started to nucleate at 500 °C (Figure 5a) into the amorphous layer, following a mechanism of homogeneous nucleation. HRTEM observations showed the presence of small zirconia crystallites, almost completely detached from the alumina surface thanks to the remaining amorphous phase. However, when a low-temperature pre-treatment (at 500 °C or 600 °C) was prolonged (up to 10 h), the ZrO_2_ crystallites were dragged by the amorphous phase flow into discrete pockets between the alumina grains, where the crystallites segregated (Figure 5b, see the arrow), leading to the aggregation and growth of the zirconia grains during sintering. On the other hand, at higher temperatures, such as 800 °C or 1000 °C, the ZrO_2_ crystallization was faster, growth predominated over nucleation, and the amorphous phase disappeared without the draining phenomenon. The result is a very different ZrO_2_ grain distribution on the alumina particle surfaces: in fact, in this case, the zirconia grains were larger, but not aggregated (see Figure 5c). This behavior was maintained during the high-temperature isothermal pre-treatments: as we can see in Figure 5d, the ZrO_2_ crystallites further grew, but were still very homogeneously distributed on the parent material. Such different sizes and distributions of the ZrO_2_ grains on the alumina particle surface gave rise to different sintered microstructures [83,85]. In fact, SEM observation carried out on ZTA composites obtained by powders calcined at 600 °C for 1 h or for 20 h showed different microstructural features. The former composite presented an even distribution of fine zirconia grains, with a narrow size distribution, inside a sub-micronic, homogeneous alumina matrix; in the latter, a less homogeneous microstructure was obtained, in which zirconia aggregates were observed.

**Figure 6:**
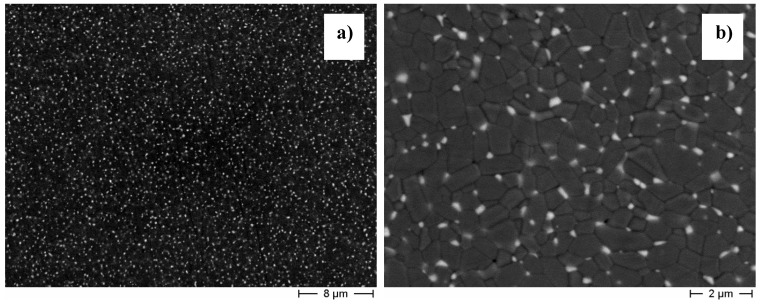
(**a**) Lower and (**b**) higher magnification FESEM images of a ZTA sintered composite.

These results show that the selection of the pre-treatment temperature is a key feature to control the phase distribution and size in the composite powders and, subsequently, in the sintered material. As an example, Figure 6 shows a typical microstructure of a ZTA composite (alumina-5 vol% ZrO_2_), obtained by pre-treating the as-dried powder at 600 °C for 1 h, after sintering at 1500 °C for 3 h. We can appreciate the very good distribution of the second phase inside the alumina matrix, and observe the lack of any alumina or zirconia aggregates. XRD analysis showed that almost all the zirconia grains crystallized under the tetragonal form, even if no phase stabilizer was added, due to the ultrafine size of the ZrO_2_ grains.

In a recent paper [86], we showed that the surface coating method is compatible with the industrial protocol commonly used for manufacturing ZTA orthopedic devices. Such a protocol implies the granulation of the composite powders, the cold isostatic pressing of the granules, their natural sintering followed by a post-HIP step, carried out to assure full densification of the components.

**Figure 7:**
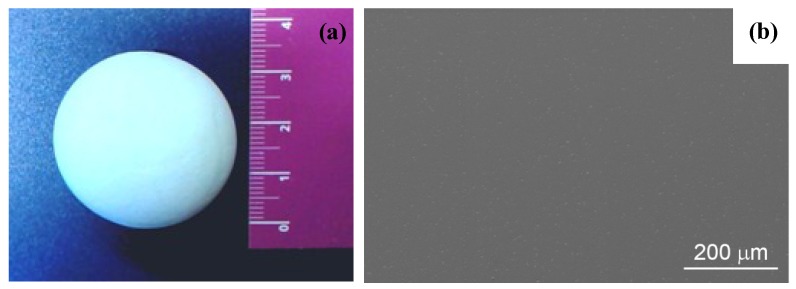
(**a**) Photograph of a ZTA femoral head prototype (diameter of 28 mm) [81]; (**b**) SEM micrograph of the cross section of the prototype, at 10 mm from the external surface of the ball [81].

According to this protocol, the granulation of the composite powder was set-up, by investigating the relationship between the rheological properties of the suspension and the morphological features of the spray-dried granules. In particular, optimal granules for pressing were obtained by controlling the viscosity of the suspension, through a flocculation step, and properly selecting type and amount of binder [86]. The prototypes were then obtained by cold isostatic pressing at 350 MPa, producing blank green bodies of spherical shape, whose mean density was about 2.43 g/cm^3^, corresponding to 58.2% of the theoretical density (TD) [83]. After a debinding thermal treatment (at 600 °C for 1 h), samples were pressureless sintered at 1500 °C for 3 h, reaching a mean fired density of 99.3% TD. The post-HIP step, carried out at 1520 °C for 2 h under a pressure of 190 MPa, allowed the bodies to achieve full densification. In Figure 7a, the photograph of one of the femoral head prototypes produced (diameter of 28 mm) is shown [83].

One sample was cut along its cross section: SEM observations, as well as Vickers hardness and fracture toughness measurements, were performed at increasing distances from the external surface of the ball. As an example, in Figure 7b the microstructure of the material at 10 mm far from the external surface is depicted, showing that it was fully dense, highly homogeneous, almost defect and agglomerates-free.

In agreement with the microstructural homogeneity observed, also the mechanical properties were almost constant across the material section: for instance, the Vickers hardness at 3 mm from the external surface was 1956.5 ± 80.6 HV and 1946.0 ± 44.8 HV at the two diametrically opposite sides. In the center of the specimens the measured hardness was 1969.5 ± 82.9 HV, showing almost constant hardness inside the ceramic sphere, independently from the position. Accordingly, an almost constant *K*_I0_ was determined all along the material section, whose mean value was about 3.8 ± 0.3 MPa·m^1/2^ [83]. This value is comparable to that obtained by slip-cast pellets (3.8 ± 0.3 MPa·m^1/2^), containing the same amount of zirconia (5 vol%) [78], showing that the results obtained by slip casting for small samples (~15 mm diameter and 3–5 mm height) can be reproduced by CIP of spray dried granules, in larger spherical components.

As a conclusion, these results highlight the potential of the surface modification route, appearing as an effective tool for tailoring the microstructural features of ZTA composite powders and sintered materials, which are maintained when the process is scaled-up to a higher level (semi-industrial), in order to prepare prototypes, and when integrated into industrial manufacturing protocols of femoral heads devices.

### 4.2. Zirconia-Based Composites for Dental Applications

The results here described refer to the activities carried out in the frame of the European Project named *Longlife* (“Advanced multifunctional zirconia ceramics for long-lasting implants”, 7^th^ Framework Program), of which the aim is the production of dental and spine implants characterized by increased strength and toughness and improved ageing stability with respect to the state-of-the-art materials.

In order to reach this goal, the materials were first properly designed, in terms of composition and microstructure.

In particular, we decided to use Ce-TZP as the matrix for the composites, since it has a lower sensitivity to Low Temperature Degradation as compared to Y-TZP. However, being aware of the key role of the stabilizing oxide content on the mechanical and physical properties of the materials, composites containing different amounts of ceria were tested, ranging from 10.0 to 11.5 mol%. Finally, on the ground of the scientific literature previously discussed, two kinds of second phases were selected, characterized by different morphologies (rounded and elongated grains) each of them playing a specific role on the materials physical and mechanical properties. The α-Al_2_O_3_ rounded grains were used to retain the Ce-TZP grain growth during sintering, increasing the strength, the hardness, the wear resistance [35,36]. On the other hand, elongated strontium hexa-aluminate (SrAl_12_O_19_) grains were chosen, as they could provide additional toughening effects by crack deflection and bridging mechanisms [40].

Considering the complexity of the phase composition and architecture of the designed composites, the surface coating route was selected as preferred method for the elaboration of the composite powders. The starting raw zirconia powder was supplied by Daiichi Kigenso, Kagaku Kogio Co. Ltd. (Japan). It is stabilized by 10 mol% of ceria, has an average particle size of 0.5–1 μm (by laser diffraction method), and a specific surface area of 14.3 m^2^/g, as declared by the supplier.

De-agglomeration of the starting powder was carried out by ball-milling: aqueous suspensions, at 33 wt% solid loading, were dispersed for about 15 h by using ZrO_2_ milling spheres, lowering the starting agglomerate size from about 1 μm to about 0.4 μm. Dilute hydrochloric acid was added, to decrease the slurry pH from the starting value of about 6.5 (close to the powder isoelectric point [87]) to about 3.

In order to obtain α-Al_2_O_3_ and SrAl_12_O_19_ second phases, Al(NO_3_)_3_·9H_2_O (≥98% purity, Sigma Aldrich) and Sr(NO_3_)_2_ (≥99.0% purity, Sigma Aldrich) were selected as precursors, and added in suitable amount to produce 8 vol% of both second phases in the composite. In order to obtain different ceria contents in the zirconia lattice, ammonium cerium (IV) nitrate ((NH_4_)_2_[Ce(NO_3_)_6_], ≥98.5% purity, Sigma Aldrich), was selected as the ceria precursor. The composites will be hereafter referred to as ZA_8_Sr_8_-CeX, where X refers to the ceria mol% inside the zirconia phase. Precisely, four composites were produced, only differing on the ceria content, being X = 10.0 (*i.e.*, no extra-ceria added during synthesis), 10.5, 11.0, and 11.5 mol%.

The nitrates were dissolved in distilled water and then drop-wise added to the dispersed zirconia suspension. After mixing for 2 h, the suspension was spray-dried.

The powder was then pre-treated at 600 °C, for 1 h, in order to decompose the synthesis by products, and then thermally treated at 1150 °C for 30 min, in order to approach the crystallization temperature of the second phases [88,89]. The calcined powders were than dispersed by ball-milling, and green bodies were produced by slip casting. Samples were then sintered at 1450 °C, for 1 h, all achieving full densification.

**Figure 8:**
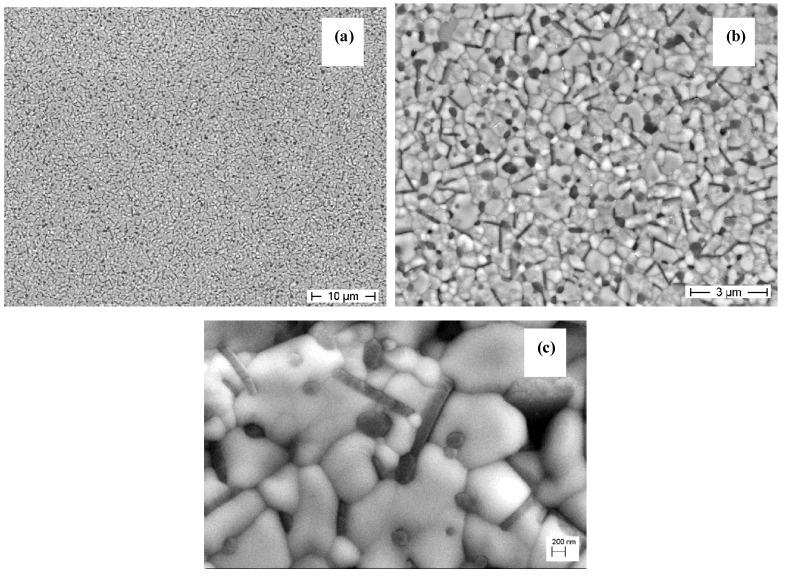
FESEM micrographs at (**a**) lower; (**b**) intermediate; and (**c**) higher magnification of ZA_8_Sr_8_-Ce11 composite sintered at 1450 °C for 1 h.

Figure 8 shows the micrograph of the sintered ZA_8_Sr_8_-Ce11 composite. In the lower magnification image (a) we can see, once again, that the surface coating route is successful in producing fully dense composites with a completely homogeneous microstructure, free from aggregates.

The higher magnification image (b) shows that all the desired microstructural features were obtained in the composite, since both rounded grains and elongated ones can be observed. Image analysis revealed an average size of 0.6 ± 0.3 μm and 0.3 ± 0.1 μm for Ce-TZP and alumina phases, respectively. The elongated (strontium aluminate) grains were characterized by a mean length of 0.6 ± 0.2 μm, and aspect ratio of 4 ± 2. The highest magnification image (c) shows that, in spite the predominant location of the second grains in intergranular position, some intra-type alumina grains can be also observed. The ceria content had no effects on the microstructure, since very similar microstructural and morphological features were observed in all composites.

**Figure 9:**
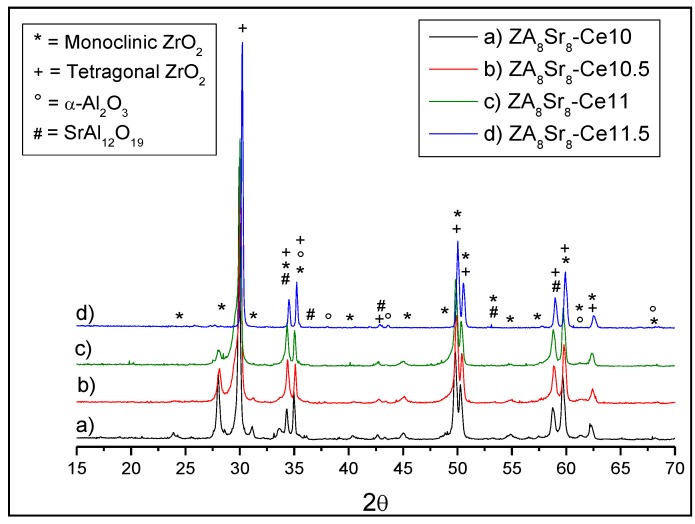
XRD patterns of ZA_8_Sr_8_-CeX composites sintered at 1450 °C for 1 h.

Contrarily, the ceria amount played a major role on other physical and mechanical properties of the materials. In fact, by XRD analyses carried out on the surface of all sintered materials, we can observe a progressive decrease of the m-ZrO_2_ phase by increasing the ceria amount (Figure 9).

The monoclinic volume fraction (*V*_m_) was calculated using the method proposed by Garvie and Nicholson [90], by which we estimated a progressive decreasing of *V*_m_ from 5 to 1, by increasing the ceria content.

**Figure 10:**
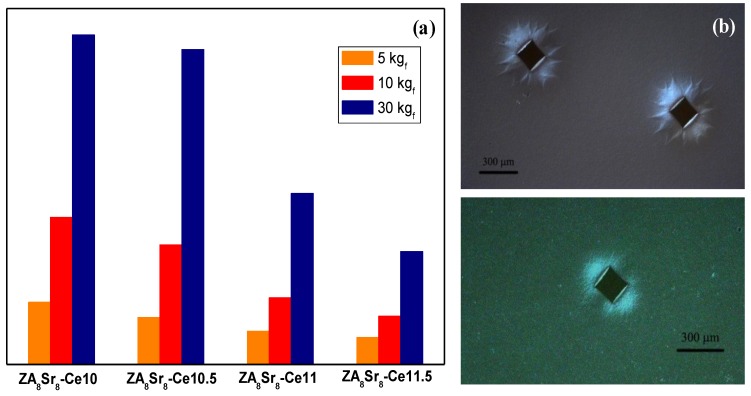
(**a**) Transformed area, for the four sintered composites, after indentation at different loads; (**b**) optical micrographs (with Nomarski contrast) of the transformed area of ZA_8_Sr_8_-Ce10 (**top**) and ZA_8_Sr_8_-Ce11.5 (**bottom**) samples.

Accordingly, the stress-induced *t-m* transformation of the sintered composites appeared to be clearly dependent on the ceria amount. This behavior was evaluated by performing Vickers indentations on the polished, sintered surfaces, at different loads (5, 10 and 30 kg_f_). Then, the size of the transformed area around each indentation was measured by means of optical microscopy, as reported in Figure 10. As expected, for all the samples, the transformed area increased by increasing the applied load. In addition, moving from the sample with the lowest ceria content (ZA_8_Sr_8_-Ce10) to the highest amount (ZA_8_Sr_8_-Ce11.5), the transformed area decreased. In fact, the higher zirconia stabilization degree in this last sample accounts for its lower transformability.

In the same figure, the optical micrographs of ZA_8_Sr_8_-Ce10 and ZA_8_Sr_8_-Ce11.5 indented samples are shown. The brighter zone around the indentation is associated to the zone in which the tetragonal to monoclinic transformation took place, providing a volume increase. In addition, such images show a different morphology of the transformed zone, depending on the ceria content. In fact, smaller round-shaped transformed areas were observed in ZA_8_Sr_8_-Ce11.5 sample, while large number of deformation branches, which radially propagated from the indentation area were observed in ZA_8_Sr_8_-Ce10 material. This last morphology evidences the autocatalytic nature of the *t*-*m* phase transformation of zirconia, meaning that once initiated, it can stimulate further transformation to propagate rapidly over an extended region.

These results have a key importance because they clearly demonstrate that the surface coating route allows an effective tuning of the ceria amount inside the zirconia lattice, where very small increases in the ceria content (of 0.5 mol%) can give rise to completely different behaviors.

A paper reporting the overall mechanical properties (flexural strength, fracture toughness, Vickers hardness) and ageing behavior of these composites is currently in progress. The here reported aims to highlight the effectiveness of the surface coating route in the elaboration of composite materials with features corresponding to the designed ones. The process here presented allows in fact the production of complex systems, leading to the fine and simultaneous control of many parameters, such as the cerium oxide stabilizer content in the zirconia lattice, the chemical compositions of the phases, their morphology, the microstructure, the final properties. For this reason, this process represents a significant advance over the existing technologies, being hopefully the next steps in its further development and scaling-up even at the industrial and clinical scale.

## 5. Conclusions

Since the early hip replacements in the 1960s, there is a continuous search for biomedical ceramic implants characterized by increasingly higher mechanical properties, reliability and *in vivo* stability.

This has allowed a constant spread of new materials, particularly focusing on composite and nanocomposite structural ceramics, where the design of new compositions and architectures seems the key to further enhance the material characteristics. The challenge is tailoring the micro- and nanofeatures in the composite structures, through a careful control of any step of manufacturing, being the synthesis of the composite powders the first and fundamental step of this processing chain.

In this frame, we have presented here an innovative elaboration method, named “surface coating route”, to process ceramic nanocomposite powders, developed in the recent years, but already demonstrating its feasibility to develop complex structures suitable for biomedical applications.

The method is illustrated into two case studies, the former concerning ZTA materials for orthopedic applications, the latter zirconia-based composites for dental implants. The two examples well prove the capability of the process to simultaneously and perfectly tailor all the compositional and microstructural features in the composite structures, leading to the fine control of the oxide stabilizer content in the zirconia lattice, the chemical composition of the phases, their size and morphology, and the overall microstructure.

Such careful tailoring of the microstructural features allows yielding good and constant mechanical properties inside the biomedical components, showing that the surface coating method can well integrate in the industrial manufacturing protocols of biomedical devices, potentially becoming an evaluable alternative to the current synthesis process already used at a clinical and industrial scale.
